# EHMTI-0318. The place of corticosteroids in migraine attack management: systematic review and critical appraisal

**DOI:** 10.1186/1129-2377-15-S1-G42

**Published:** 2014-09-18

**Authors:** YW Woldeamanuel, AM Rapoport, RP Cowan

**Affiliations:** 1 Neurology and Neurological Sciences, Stanford University School of Medicine, Stanford, USA; 2 The David Geffen School of Medicine, University of California in Los Angeles, Los Angeles, USA

## Introduction

Headaches recur in up to 87% of migraine patients visiting the emergency department (ED), making ED recidivism a management challenge.

## Aims

We aimed herein to determine the role of corticosteroids in the acute management of migraine in the ED and outpatient care.

## Methods

A PubMed search was employed for Clinical Studies and Systematic Reviews on the PubMed Clinical Queries tool combining the terms ‘migraine’ and ‘corticosteroids’ from 1980 until May 1, 2014.

## Results

Twenty-two studies (n=2203, 50% ED-based, 64% randomized-controlled) and four systematic reviews were included. International Classification of Headache Disorders criteria were applied in 68%. Twenty-one studies indicated observed outcome differences favoring benefits of corticosteroid administration. Median absolute risk reduction was 30% (range 6 - 48.2%) and 11% (6 - 48.6%) for 24- and 72-hour headache recurrence, respectively. Parenteral dexamethasone was the most commonly (65%) administered steroid, at an average single dose of 12.8 mg (range 4 - 24 mg). All meta-analyses revealed efficacy of adjuvant corticosteroids to various abortive medications – indicating generalizability. Adverse effects were tolerable. Higher disability, status migrainosus, incomplete pain relief, and previous history of headache recurrence predicted outcome favourability.

## Conclusions

Our literature review suggests that with corticosteroid treatment, recurrent headaches become milder than pretreated headaches and later respond to nonsteroidal therapy. Single-dose intravenous dexamethasone provides reasonable option for managing resistant, severe, or prolonged migraine attacks; recommendations include 6-8 administrations per year with follow up of adverse effects.

No conflict of interest.

